# Mini‐invasive technique for peroral endoscopic myotomy in adult patient: “Slim peroral endoscopic myotomy” procedure

**DOI:** 10.1111/den.14494

**Published:** 2022-12-27

**Authors:** Francesco Azzolini, Francesco Vito Mandarino, Silvio Danese

**Affiliations:** ^1^ Division of Gastroenterology and Gastrointestinal Endoscopy San Raffaele Hospital Milan Italy

## Abstract

Watch a video of this article.

## Brief explanation

A 71‐year‐old female patient, with type III achalasia and Eckardt score 5 (dysphagia 3, regurgitation 1, chest pain 1, weight loss 0) was referred to our center. We performed an upcoming esophageal peroral endoscopic myotomy (POEM) technique, using pediatric gastroscope (EG16‐K10, 5.4 mm insertion tube diameter, 2.0 mm working channel diameter; Pentax Medical, Tokyo, Japan) for most of the procedure, with the aim of experiencing a less invasive based‐long‐myotomy procedure, in terms of submucosal tunnel width.

**Figure 1:**
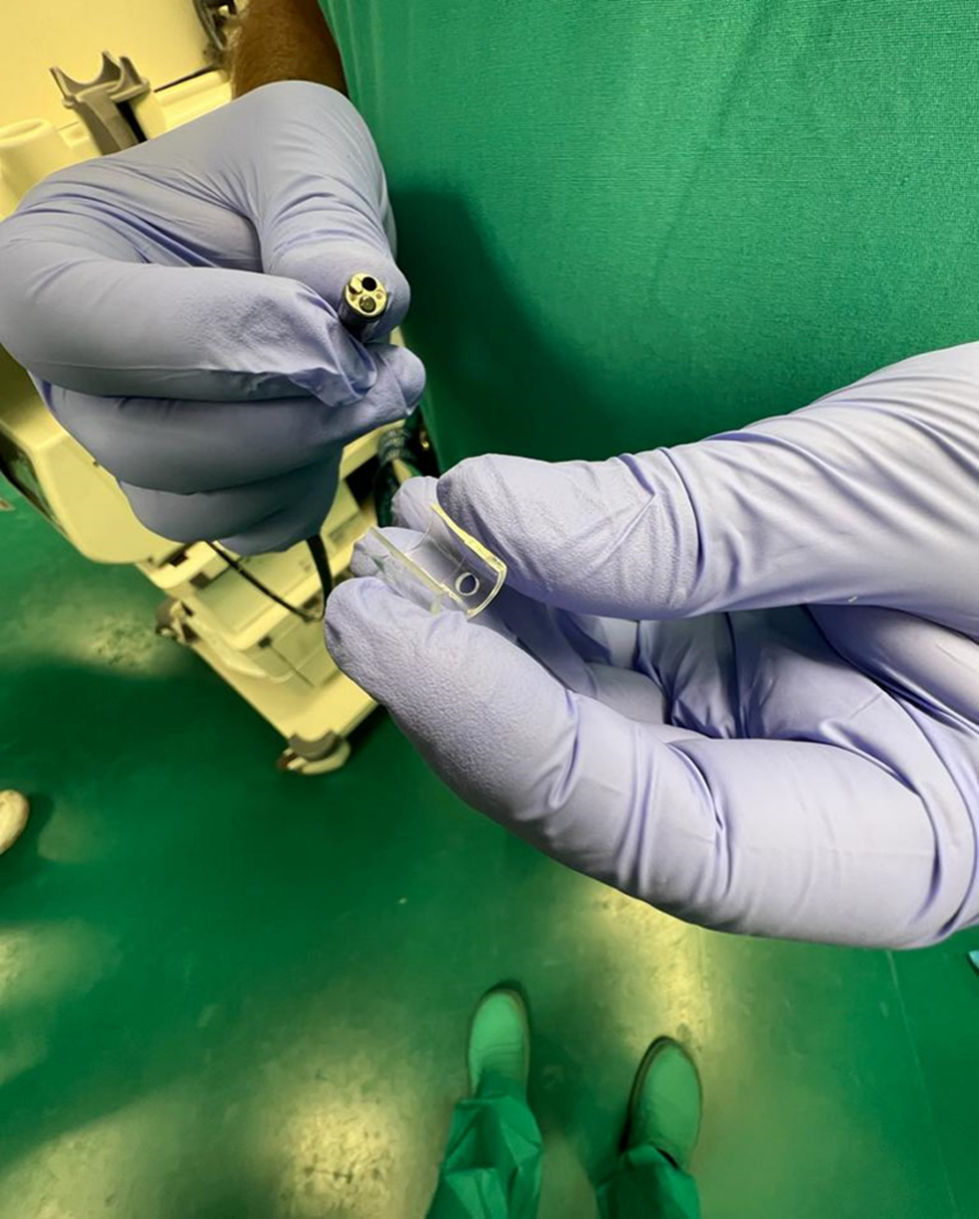
The manufactured cap attached on the tip of the slim scope.

**Figure 2:**
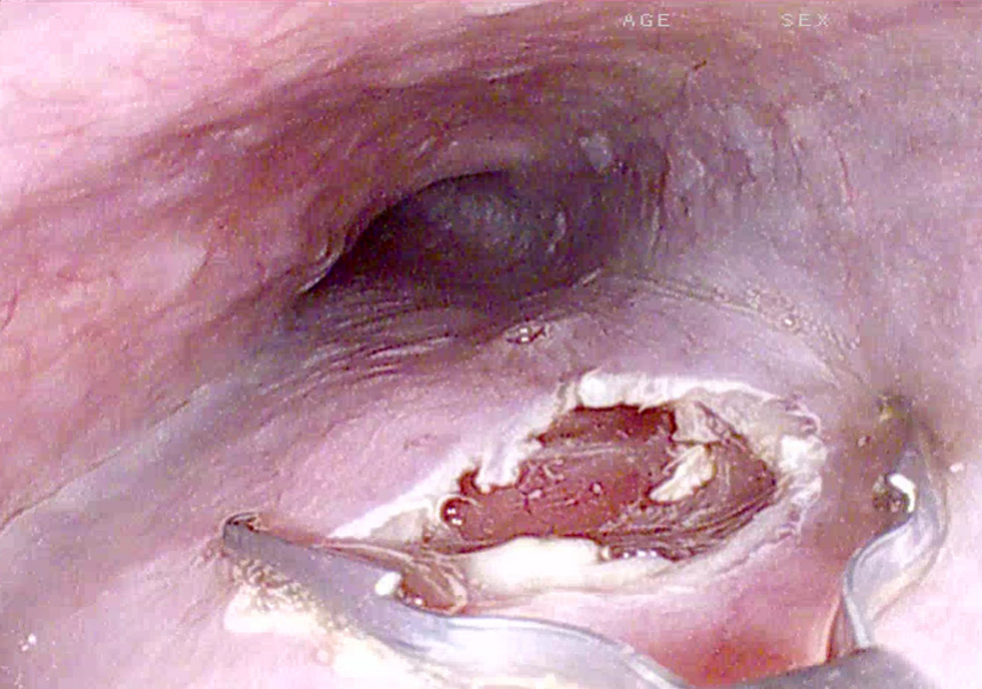
The apposition of a single clip to close the mucosal entry.

The slim scope was fitted with a manufactured‐in‐size cap (Steris Healthcare, Mentor, OH, USA) (Fig. 1). A submucosal cushion at 30 cm from incisor teeth, with solution of glycerol, epinephrine and carmine indigo injected through a pre‐cut needle was created. The mucosal opening was performed using a monopolar coagulation probe (Maslanka, Tuttlingen, Germany). The submucosal tunneling was carried out dissecting with the probe up to 5 cm beyond the cardias. During tunnelization, the same probe was used to stop an episode of arterial bleeding. Then, we performed myotomy, using Knife‐Snake papillotome (G‐FLEX, Nivelles, Belgium), closing the mucosal entry, with a single clip, using a standard gastroscope (Fig. 2). The procedure lasted 43 min.

After 1 month, the patient reported symptoms improvement (Eckardt score 2: dysphagia 1, regurgitation 0, chest pain 1, weight loss 0).

To best to our knowledge, this is the first case of “slim POEM” on an adult patient (Video S1). Coelho Conrado *et al*.^1^ have already described a similar technique on a pediatric patient.

Advantages of the slim scope approach could include lower risks of intra‐procedural complications and cost, and minimally invasive tailored myotomy (lower risk of long‐term gastroesophageal reflux with same clinical efficacy?), compared to the standard technique. However, the unavailability of feasible devices through 2.0 mm working channel‐scope for management of potential massive bleeding (i.e., Coagrasper forceps) may represent the most critical issue and could encourage their development.

## CONFLICT OF INTEREST

Author S.D. has served as a speaker, consultant, and advisory board member for Schering‐Plough, AbbVie, Actelion, Alphawasserman, AstraZeneca, Cellerix, Cosmo Pharmaceuticals, Ferring, Genentech, Grunenthal, Johnson and Johnson, Millenium Takeda, MSD, Nikkiso Europe GmbH, Novo Nordisk, Nycomed, Pfizer, Pharmacosmos, UCB Pharma and Vifor. The other authors declare no conflict of interest for this article.

## Supporting information


**Video S1** Peroral endoscopic myotomy procedure performed with a slim pediatric gastroscope.
