# Supplementary material for: Mini‐invasive technique for peroral endoscopic myotomy in adult patient: “Slim peroral endoscopic myotomy” procedure
Source: Dig Endosc. 2022 Dec 27;35(3):e37–8. doi: 10.1111/den.14494 (PMC12136272; doi:10.1111/den.14494)
Supplement: Supplementary file 1 — Video S1 Peroral endoscopic myotomy procedure performed with a slim pediatric gastroscope. [file DEN-35-e37-s001.zip › den14494-sup-0001-VideoS1/den14494-sup-0001-VideoS1.docx]

**Video legend**

POEM procedure performed with a slim pediatric gastroscope.

**Text:** A 71-year-old female with diagnosis of type III achalasia. At diagnosis, Eckardt score: 5 points (dysphagia 3, regurgitation 1, chest pain 1, weight loss 0)// A novel approach of Peroral Endoscopic Myotomy was performed, using a slim gastroscope (5.4 mm outter diameter and 2.0 mm working channel) either for mucosal entry, submucosal tunneling and for myotomy// A manufactured cap was tailored on the tip of the slim scope. Afterwards the submucosal injection was performed with a pre-cut needle//Submucosal cushion//Mucosal entry and subsequent tunnelization were performed using a monopolar coagulation probe//Mucosal entry//Tunnelization//The myotomy was performed using a papillotome//Myotomy with papillotome//Completed myotomy//Passage through the floppy cardias//Single clip apposition// Post-procedural controlo gastrographin study showing no leaks and easy passage of the contrast through the cardias// The patient was discharged on the third post-operative day (POD). At 1 month evaluation symptoms significantly improved (Eckardt score 2: dysphagia 1, regurgitation 0, chest pain 1, weight loss 0).
